# Analysis of the influencing factors of vitality and built environment of shopping centers based on mobile-phone signaling data

**DOI:** 10.1371/journal.pone.0296261

**Published:** 2024-02-15

**Authors:** Xiaohe Bai, Min Zhou, Weiming Li

**Affiliations:** 1 Faculty of Innovation and Design, City University of Macau, Macau, China; 2 Dongguan Urban Construction Planning and Design Institute, Dongguan, China; 3 Faculty of Horticulture and Landscape architecture, Yangzhou Univeristy, Yangzhou, China; Sabancı Üniversitesi, TURKEY

## Abstract

Nowadays, shopping centers not only provides commercial function but also serve as a public space. In this article, we take Nanshan district of Shenzhen as an example, based on the characteristics information of people activities provided by Mobile-phone Signaling Data, using the standard deviation ellipse method to classify the direction of people in shopping centers, and then applying the entropy weighting method to analyze the vitality factors of shopping centers from three perspectives: visitors’ density, revisit rate, and the average length of stay. Finally, we analyzed the influence factors of the surrounding built environment based on correlation analysis to discuss the results with field survey data. The results show that (1) shopping centers in Nanshan District are classified into wide-area type and geo-regional type according to the gathering of visitors. The shopping centers with high comprehensive vitality are basically wide-area type. (2) The factors influencing the vitality of shopping centers are different between wide-area type and geo-regional type. The vitality of wide-area type is mainly influenced by the traffic accessibility and whether they are located in adjacent to large public spaces such as squares and green public; the vitality of geo-regional type shopping centers is mainly influenced by the number of people within a 15-minute walking circle, and the high-vitality of geo-regional shopping centers are generally located in densely populated areas.

## 1. Introduction

The construction of commercial complex is currently in full swing in first-tier cities and is accelerating its spread to second-tier and third-tier cities. According to Win Business Website, a total of 84 commercial complexes opened in China in Q3 of 2020(July-September), nearly 7.95 million square meters. The four first-tier cities, namely Beijing, Shanghai, Guangzhou and Shenzhen, opened a total of 10 projects in Q3 of 2020, with Shenzhen having the most. (Win Business Website is based on serving commercial real estate, with the largest brand merchant expansion site library and the largest commercial real estate project library in China.)Among the opened projects are mainly shopping centers, medium-sized shopping centers in the range of 50,000 to 100,000 square meters (excluding 100,000 square meters) are the mainstream, accounting for 40% of the total, with 34 projects.

The research trends on shopping centers over the last five years have four main directions: using multi-source big data to analyze the impact of shopping centers on the spatial structure of urban business; the connection between shopping centers and urban transit; the impact of shopping centers on consumers’ behavior and its mechanisms; the evaluation of the attractiveness of shopping centers themselves; as well as the analysis of related influencing factors. The first research direction mainly uses POI data and Baidu Heat Map Data, to conduct survey along with other commercial facilities in the city [1–3]. The second direction is that more and more researchers in China are considering shopping centers with urban transit, in order to analyze the relationship between them [4,5]. Moreover, they investigated the features of the interface between shopping centers and the traffic station in terms of spatial synergy [6,7]. The third direction usually is to study the influence of shopping centers’ location on consumer behavior and its mechanisms, in order to study the temporal and spatial differences in the behavior of different types of individuals [8], aiming to optimize shopping center’s location. The last direction is the appraisal of the center’s attractiveness and the examination of the relevant influencing elements [9], which are gradually diversifying, the selection of consumer groups is also getting more precise [10], and in addition to studies on undifferentiated groups [11], there has been a recent consideration from the standpoint of older individuals [12]. While few research have categorized shopping centers in different types in depth in order to better analyze their spatial characteristics.

Jane Jacobs initially highlighted the development of urban vitality in her book The Death and Life of Great American Cities [13], in which she stated that ’life’ in city is formed of the actions of people in city streets. Since then, the study of urban vitality started from the ’street’. As streets’ metrics can depict physiognomy in detail, in that case, street vitality affected by street is a concrete manifestation of urban vitality. Researches mainly focused on the influencing factors [14], mechanisms [15], and visual characteristics [16]. While, not only streets, but also parks, squares, neighborhoods, and shopping centers, have become popular open space for people to gather on a regular basis. Among the eight strategies for creating the vitality of contemporary urban open space, it is mentioned that shopping centers in cities that stimulate activities and events are important factors for "vitality" [17]. At present, research on urban vitality is primarily focused on two aspects. The first aspect focused on urban public space from a macroscopic perspective, with an emphasis on the development of a vitality rating system for public space as a whole and taking the total vitality of urban space into account [18]. The second aspect is to choose the certain types of open space. With the increase in the diversity of people’s needs for social activities, there are more and more relevant research on specific types of public space as the object of vitality evaluation, such as parks, squares, scenic spots, etc., which are biased towards microscopic details [19], put more attention on the interaction between individuals and urban open space [20], aiming to figure out the factors that could influence vitality. At present, the objects of vitality research are mostly focus on outdoor area or open space which belongs to public welfare. However, shopping centers have gradually developed into large integrated commercial complexes due to their integration function of shopping, entertainment, leisure, culture and appreciation, which not only provide shopping function but also assume the function of public space. While previous studies have not studied shopping centers from the perspective of public space. Although shopping centers are not public welfare properties, they also undertake the function of public entertainment and are part of public space. From this view, the study aiming to conduct vitality of shopping centers has its unique practical meaning.

In this article, we are aiming to quantify the vitality of shopping centers, taking Nanshan District of Shenzhen as the study area, and extracting the shopping centers in Nanshan District of Shenzhen according to the POI data of Shenzhen in 2018, as well as combined with the data of Win Business Network which is the largest online information library of branded merchant expansion sites and commercial real estate projects in China for query and comparison. Finally, counting a total of 29 shopping centers in Nanshan District of Shenzhen by the end of 2018 [2], of which twelve objects were chosen. Meanwhile, based on the previous research, Mobile-phone Signaling Data(MSD) was selected to measure the vitality of shopping centers [21]. Firstly, we have divided shopping centers into wide-area and geo-regions types based on the source of applicable population by using the Standard Deviational Ellipse method. Wide-area type refers to the dispersed distribution of people visiting shopping centers, with services aimed at a wider range, the larger area of the standard deviation ellipse. Geo-regions type refers to the concentrated distribution of people visiting shopping centers, with services targeting a smaller range, the shorter the short axis of the standard deviation ellipse. Then based on the different types, the study analyzes the vitality characteristics of shopping centers and the factors influencing the surrounding built environment from the following three perspectives: pedestrian density, revisit rate, and the average length of stay. The rest of this study is structured as follows. Section 2 presents the study data and methodology. Section 3 discusses the selection of vitality indicators, the location and spatial characteristics of vitality and their vitality ranking. Section 4 reveals the mechanisms and in corresponding to the results of the field research demonstration. Lastly, Section 5 provides a concluding commentary and discussion.

## 2. Materials and methods

### 2.1 Data sources

The residential population statistics in Nanshan District, Shenzhen is derived from two sources. The first is residential building data from Nanshan District, Shenzhen in 2018, which depicts the residential population trend through residential building data. The other one comes from the Unicom Smart Footprint platform, which identifies the residential population by identifying the residential locations of Unicom users, whose residential locations are observed from 21:00 to 8:00 the next day, and the number of seconds that a user was observed daily during the observation period is accumulated monthly and ranked, with the highest ranking being the residential places of users. This two information could corroborate each other and improve the credibility of the Unicom data.

The mobile phone signaling data of visitors to shopping centers in Nanshan District comes from the mobile phone signaling data of anonymous Unicom users in Shenzhen, with a spatial accuracy of 250M, obtained from the Unicom Smart Footprint platform. Wisdom Footprint has pre-processed the data of mobile phone users, which is obtained through SQL code according to the research requirements. The visitor density of shopping centers is estimated based on the total number of visits to the center in a week, and a simple statistical analysis was conducted. The weekly revisit rate of shopping centers is measured using visit records from 60k users to shopping centers during a one-week period (19.10.2019–25.10.2019). (The revisit rate of 60k users was collected by the Unicom platform. The collection principle is the number of times the same signal repeatedly appears within a given time. If the number of occurrences is ≥ 2, it will be considered a revisit.)The dynamic performance of shopping centers is the average residence time of Unicom users.

The isochronous circle data for Nanshan district shopping centers is separated into two categories: walking isochronous circle and bus isochronous circle. Walking isochrones circle are obtained via the MAPBOX open platform with 15 minutes, whereas bus isochrones circle is obtained via Gaode Map open platform with 45 minutes.

### 2.2 Research methodology

#### 2.2.1 Standard deviational ellipse

Lefever invented the standard deviational ellipse in 1926 [22], and it is widely utilized as a spatial statistical tool to disclose the spatial distribution and spatial and temporal evolution of geographical elements from a global and spatial perspective at present. The standard deviational ellipse is employed in this paper to visualize the aggregation properties of the distribution of dwellings of shopping center visitors. The exact formula is as follows:

Let the point coordinates of the residence of all visitors be (x1, y1),(x2, y2),…,(xn, yn), then the standard variance ellipse points to tanθ, maximum standard deviation distance σx is the length of the long axis of the ellipse, minimum distance σy is the length of the short axis of the ellipse [23], calculated by the following formula:

$$\tan\;\theta =\frac{\sum_{i=1}^{n}{\left(x_{i}-\bar{x}\right)}^{2}-\sum_{i=1}^{n}{\left(y_{i}-\bar{y}\right)}^{2}+\sqrt{{\left[\sum_{i=1}^{n}{\left(x_{i}-\bar{x}\right)}^{2}-\sum_{i=1}^{n}{\left(y_{i}-\bar{y}\right)}^{2}\right]}^{2}+4{\left[\sum_{i=1}^{n}(x_{i}-\bar{x})\sum_{i=1}^{n}\left(y_{i}-\bar{y}\right)\right]}^{2}}}{2\sum_{i=1}^{n}\sum_{i=1}^{n}(x_{i}-\bar{x})\sum_{i=1}^{n}\left(y_{i}-\bar{y}\right)}$$ (1)

$$\sigma_{x}=\sqrt{\sum_{i=1}^{n}\frac{{\left[(x_{i}-\bar{x})\cos\;\theta -(y_{i}-\bar{y})\sin\;\theta \right]}^{2}}{n}}$$ (2)

$$\sigma_{y}=\sqrt{\sum_{i=1}^{n}\frac{{\left[(x_{i}-\bar{x})\sin\;\theta -(y_{i}-\bar{y})\cos\;\theta \right]}^{2}}{n}}$$ (3)

Where $\bar{x},\;\bar{y}$ is the average of the x-coordinate and y-coordinate values of the settlement respectively; θ is the directional angle of rotation. Tanθ is between 0 and 1, with 0 = the roundest and 1 = the flattest. The size of the standard deviational ellipse represents the extent of the spatial distribution of the residential regions of shopping center visitors, with smaller size being more concentrated and bigger size being more discrete. The ellipse’s long half-axis represents the direction of resident distribution, while the short half-axis reflects the extent of residence dispersion. The larger the gap in values between the long and short half-axes (the greater the flatness), the more apparent the direction of the residency distribution. The shorter the short semi-axis, the more centripetal the residences are and they are more close to the service area, indicating a higher concentration of shopping center visitors. Conversely, the longer the short semi-axis, the greater the dispersion of the residences, indicating a higher dispersion of shopping center visitors.

#### 2.2.2 Entropy weight method

The Entropy Weight Method(EWM) is used to calculate the shopping center’s vitality after combining all of the vitality indicators. The EWM determines the weight of each indicator based on the degree of disorder, the larger degree of disorder of the indicator, the higher its impact on the comprehensive evaluation, the greater the weight, this objective weighting method can effectively avoid the bias caused by human factors [24]. The Mpai data science platform was utilized in this work to calculate the weights of the aforementioned vitality indicators, and the weights were then used to combine the vitality indicators into a composite vitality value. The following is the calculating formula:

$$Vitality\;value=\frac{Density*b1+Visit\;rate*b2+A*b3}{3}$$ (4)

In this formula, “Vitality” is the shopping center’s comprehensive vitality score, between 0 and 1, with 0 = the highest vitality and 1 = the lowest vitality. “Density” reflects Visitor density. “Visit rate” represents the revisit rate. “A” represents the average length of stay. The weights of Visitor density, revisit rate, and average length of stay determined by the EWM are denoted by “b1”, “b2”, and “b3”respectively. Because the molecules vitality values are above three quantities. So the final normalization is divided by 3.

#### 2.2.3 Correlation analysis

On the basis of relevant literature research, we use a linear regression model to analyze the influencing factors of shopping centers [25].


Y=βX+ε (5)

“Y” is the vector of dependent variables; “X” is the matrix of independent variables; “β” is the coefficient of independent variables; and “ε” is the error vector of the normal distribution.

## 3. Results

### 3.1 Vital characteristics of shopping centers in Nanshan District

#### 3.1.1 Shopping center categories

**Fig 1:**
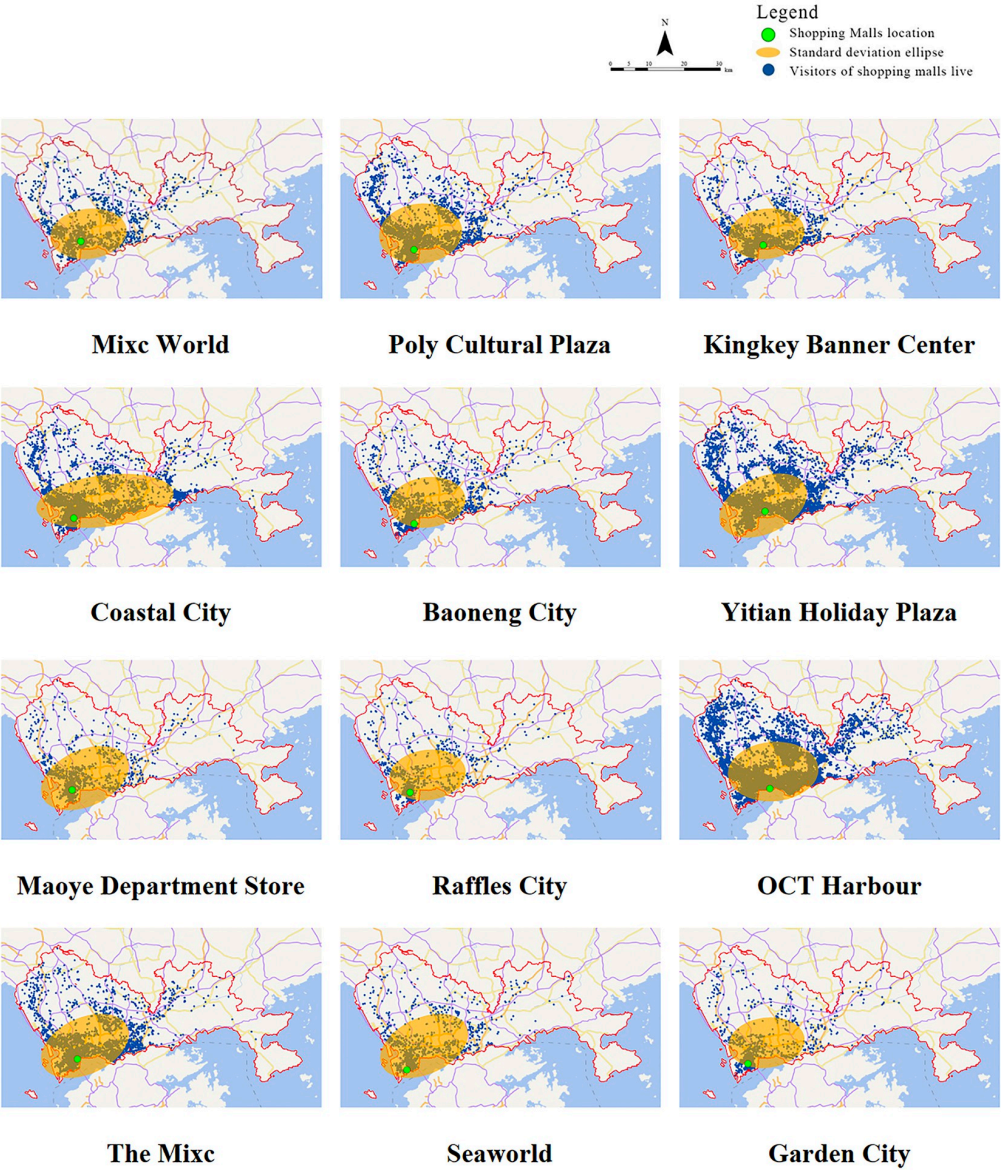
Standard deviational ellipse of the distribution of visitors to each shopping center.

Shopping centers are classified based on the origin of the people who use them, and the standard deviational ellipse analysis is used to analyze the location of visitors to each shopping center in Nanshan District (Fig 1). The results of the standard deviational ellipse reflect the aggregation and direction of the distribution of visitors’ places of residence from macro perspective.

**Fig 2:**
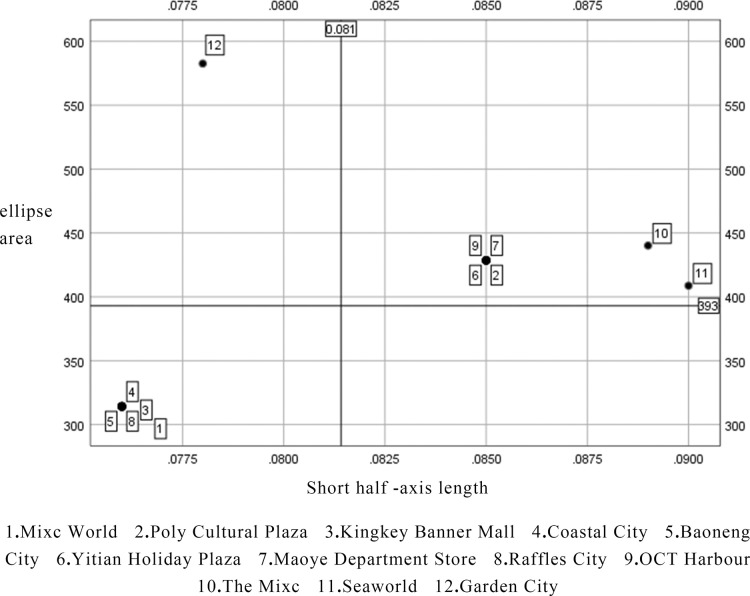
Standard deviational ellipse area of the distribution of the place of residence of visitors to each shopping center in Nanshan District in relation to the length of the short semi-axis.

The greater the size of the standard deviational ellipse, the broader the features of each shopping center, and the shorter the short axis of the standard deviational ellipse, the more geo-regional the characteristics of each shopping center. To continue the analysis of the wide area and geo-regional area characteristics of each shopping centers, using SPSS software, the length of the short axis of the standard deviational ellipse of each shopping center is the X-axis, the area of the standard deviational ellipse is the Y-axis, and the mean value of the length of the short axis and the area of the ellipse is the division point of the XY-axis. The specific results are as follows (Fig 2): The six shopping centers with the widest range of characteristics are Seaworld, Maoye Department Store, The Mixc, Yitian Holiday Plaza, OCT Harbour and Poly Cultural Plaza, this type of shopping centers have a wide range of services and a discrete distribution of service population; the five most geographically diverse shopping centers are Baoneng City, Garden City, Kingkey Banner Center, Raffles City and Mixc World. The service scope of this type of shopping centers is relatively scenter and the service population is distributed and concentrated, in other words, it serves a relatively significant number of people in its immediate surroundings. While there is one object that is both wide and geo-regional, namely Coastal City, which serves people at a distance and has a major clustering impact on the surrounding population. (Table 1)

**Table 1:** Nanshan District Shopping Center type classification table.

Type	Shopping Center
Geo-regional type	Raffles City, Garden City, Baoneng City, Mixc World, Kingkey Banner Center
Wide-area type	Seaworld, Maoye Department Store, Poly Cultural Plaza, The Mixc, OCT Harbour Yitian, Holiday Plaza
Mixed type	Coastal City

#### 3.1.2 Vitality indicator selection

Beginning with the concept of spatial vitality, the fundamental technical approach to studying urban spatial vitality has been describing spatial vitality around people and activities, identifying the characteristics of spatial vitality, and finding the components of vitality. Therefore, the three main perspectives of spatial vitality characterization, namely “Aggregation”, “Spatial revisit rate”, and “Activity diversity”, were chosen from the combination of existing research results (Table 2), which investigated the characteristics and factors influencing the vitality of shopping centers in Nanshan District, Shenzhen.

**Table 2:** Elements of spatial vitality evaluation.

Angle	Description
Aggregation[26–29]	Reflects the concentration of people in a space; the most intuitive representation of spatial vitality
Spatial revisit rate[30]	Reflects the multiple visits of people; the attractiveness of the space
Activity diversity[31–33]	Average length of stay of visitors, the longer the length, the more types of activity that will happen

The most representative indicators were chosen based on three perspectives, namely Visitor density, revisit rate, and average length of stay of visitors as indicators of shopping center vitality, and visitor arrival information was extracted from mobile phone signaling data.

#### 3.1.3 Vitality ranking characteristics

**Fig 3:**
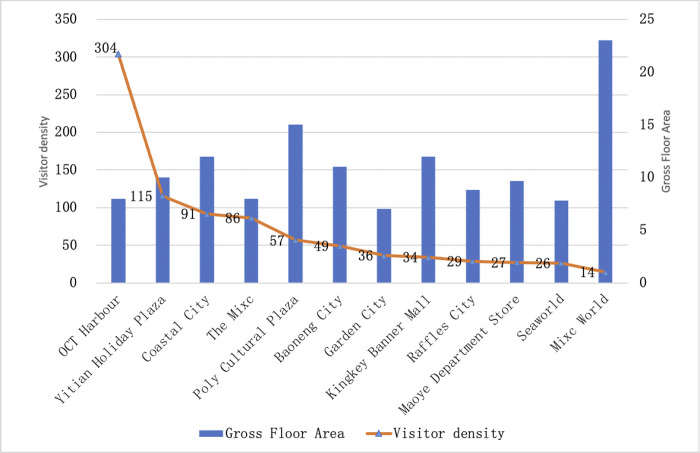
Visitor density of shopping centers in Nanshan District.

The visitor density indicator reflects the agglomeration of shopping centers and is the most intuitive indicator of vitality. The findings suggest (Fig 3) that the average visitor density of shopping centers in Nanshan District is 72, but there are significant differences in distribution, with the highest visitor density is OCT Harbour, a wide-area shopping center, and Mixc World has the lowest visitor density, a geo-regional center, with a 21.71 times range in visits per unit area and a more pronounced hierarchical character. Moreover, we found that Mixc World has the largest area while has the lowest visitor density.

**Fig 4:**
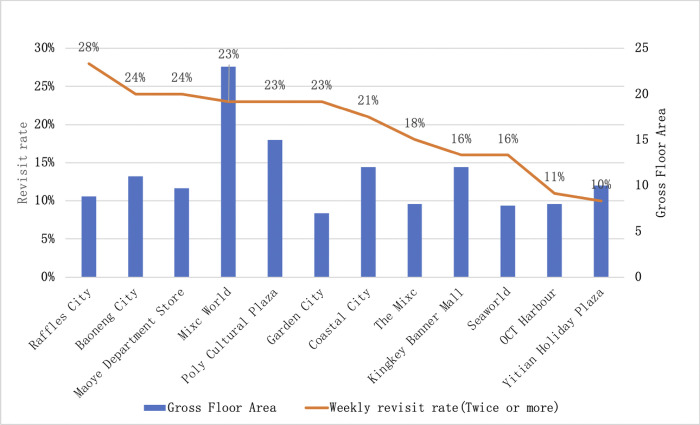
Weekly revisit rate (2 or more times) of Shopping Centers in Nanshan District, in order of rank.

The revisit rate indicator reflects a person’s repeated visit behavior and reflects the attractiveness of shopping centers. The results show (Fig 4) that the average weekly revisit rate for Nanshan District Shopping Centers is generally 20%, but there are significant differences in the distribution, with the maximum revisit rate for Raffles City Shopping Center, a geo-regional center, and the minimum for Yitian Holiday Plaza, a wide-area shopping center; the range of shopping center revisit rates close to 2.8 times.

**Fig 5:**
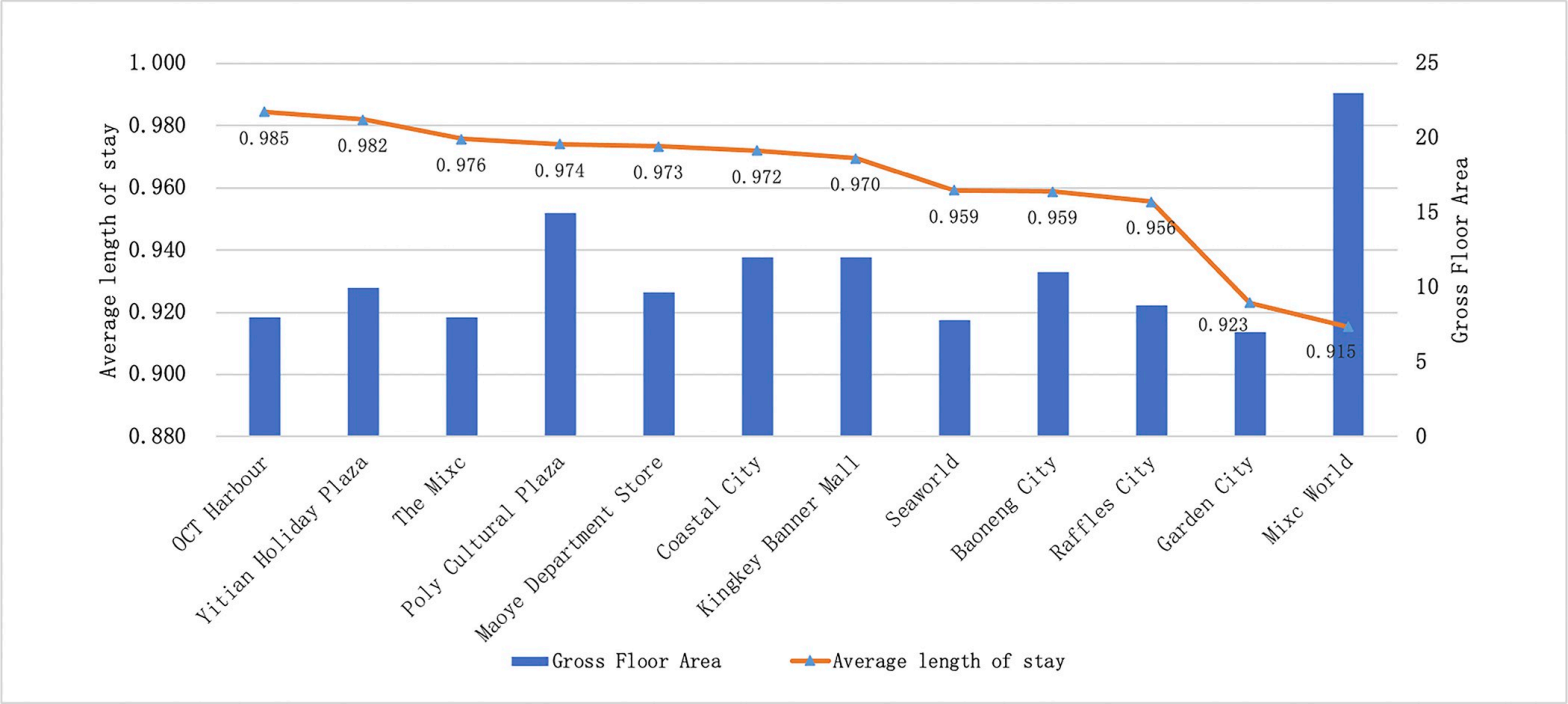
Average length of stay to Shopping Centers in Nanshan District, in descending order.

The longer the visitor flow stays, the more types of activities may occur in shopping centers, which can reflect the diversity of shopping centers. It conducted (Fig 5) that the average length of stay in Nanshan District Shopping Centers is 0.49h, with no significant difference in distribution. Wide-area shopping centers have a longer average length of stay, while geo-regional centers have a shorter average length of stay.

**Fig 6:**
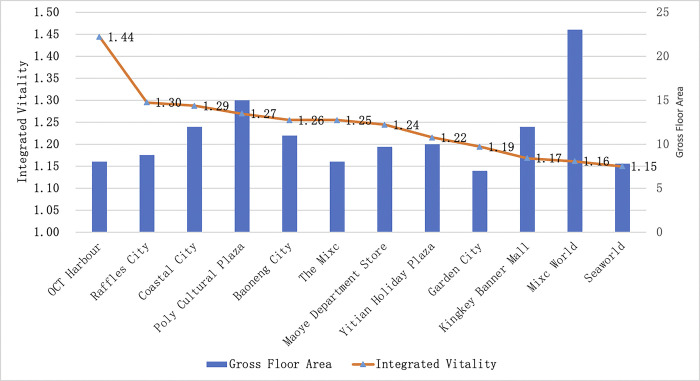
Average length of stay to Shopping Centers in Nanshan District, in descending order.

According to the EWM, the overall distribution of shopping center dynamics is more balanced (Fig 6), with OCT Harbour having the highest comprehensive vitality. It is also obvious that Visitor density has the biggest impact on a shopping center’s comprehensive vitality. Mixc World, the shopping center with the largest commercial area, except revisit rate, it has lower results in other metrics.

In general, the vitality ranking of shopping centers in Nanshan District has two characteristics. Firstly, wide-area shopping centers have high Visitor density, long average length of stay and high comprehensive vitality, while geo-regional shopping centers have high revisit rates. Secondly, there are more obvious hierarchical characteristics of Visitor density, and it has the greatest impact on the comprehensive vitality of shopping centers.

#### 3.1.4 Spatial distribution characteristics of dynamic performance

**Fig 7:**
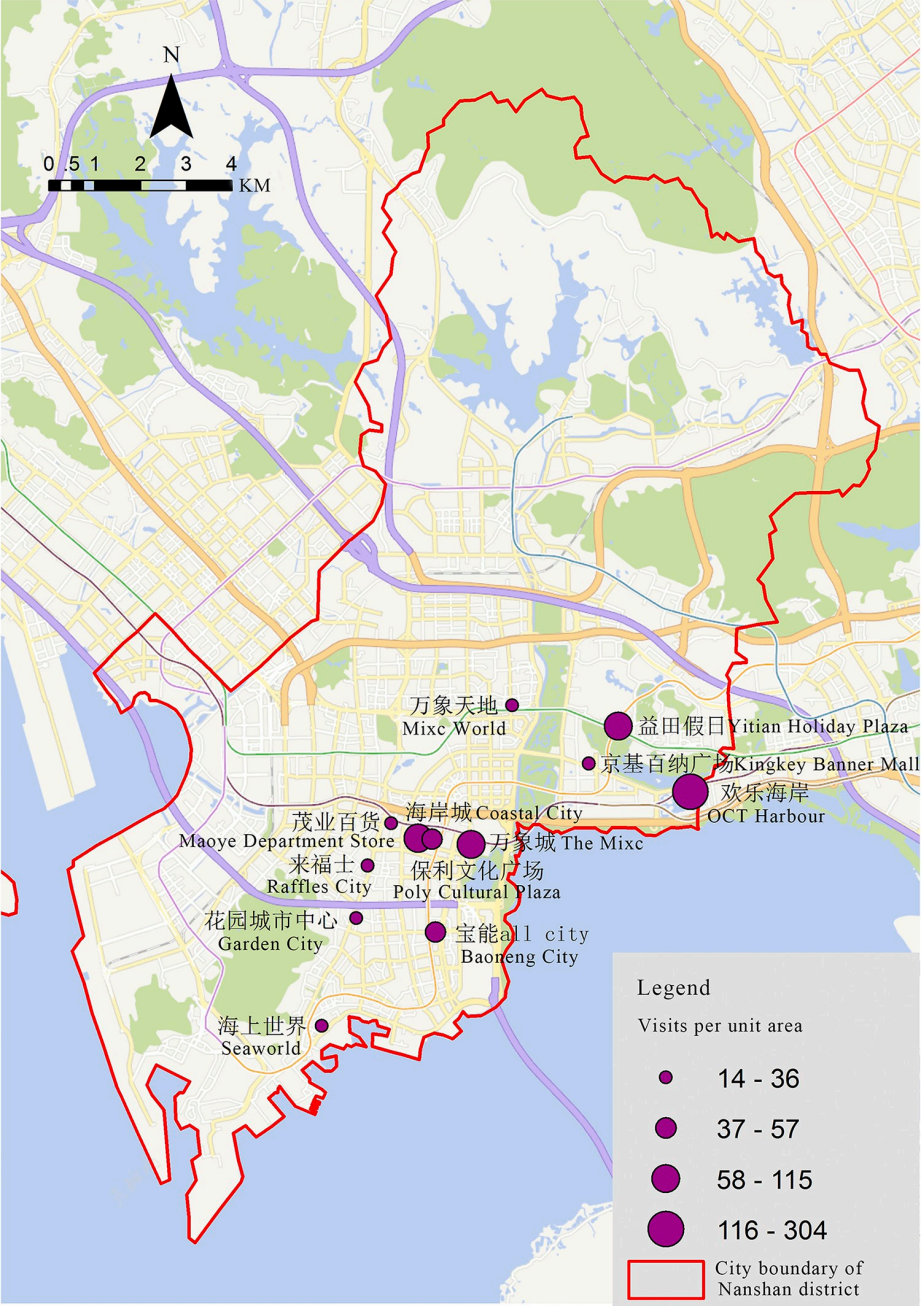
Spatial distribution of visits per unit area in shopping centers in Nanshan District.

In the spatial distribution of pedestrian density in shopping centers, there is a substantial multi-point clustering phenomenon. The results show that (Fig 7) a high-visit rate circle was formed that Coastal City as the core, with The Mixc and Poly Cultural Plaza. The other high-visit rate circle was taken the Window of the World as the core, along with the OCT Harbour and Yitian Holiday Plaza. While, lower-visit shopping centers, such as Maoye Department Store, Raffles City, and Kingkey Banner Center Plaza, are surrounding these two circles.

**Fig 8:**
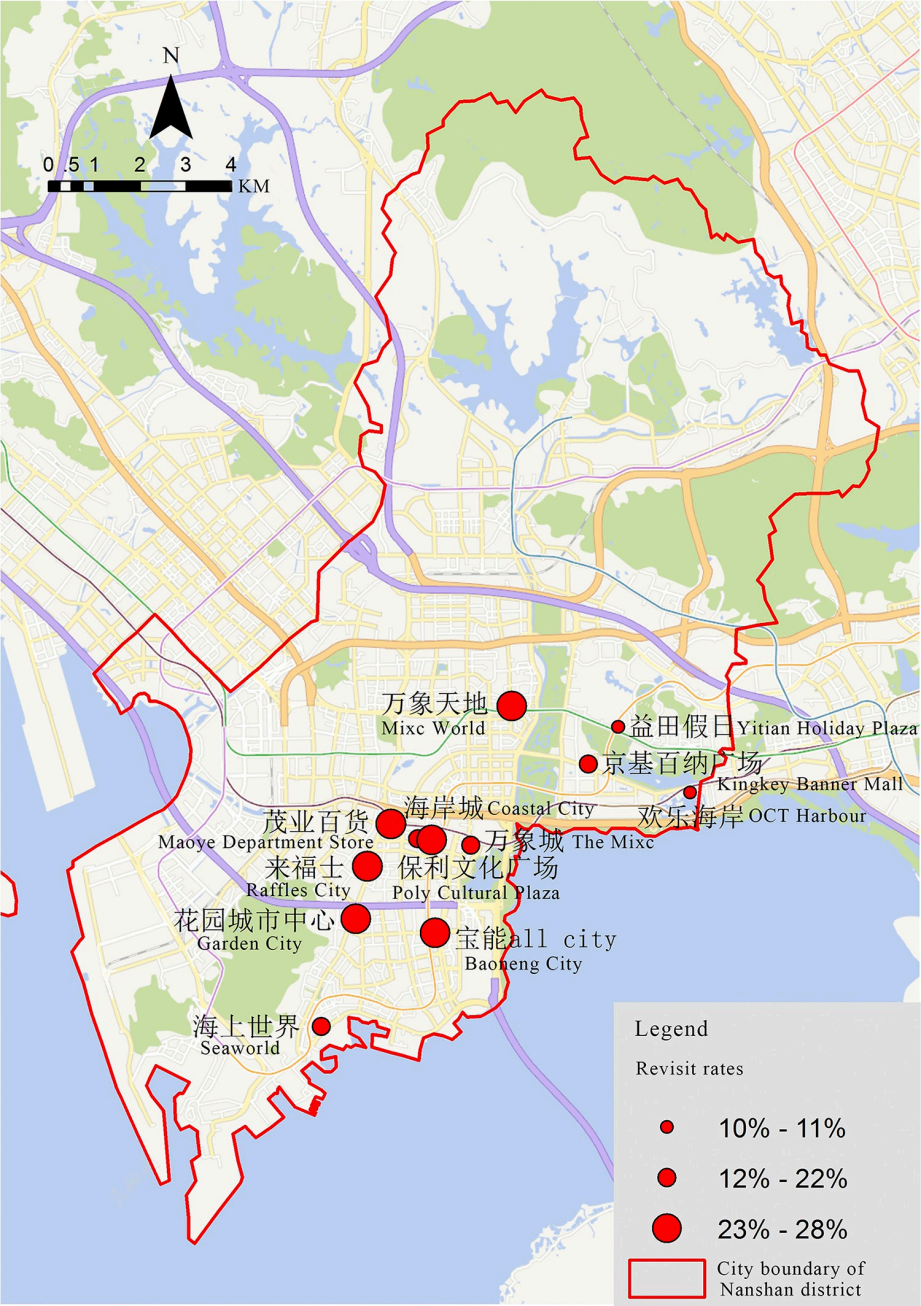
Spatial distribution of revisit rates in shopping centers in Nanshan District.

The spatial distribution of shopping center revisit rates shows a significant single-point clustering phenomenon (Fig 8). The overall revisit rate of shopping centers is higher in the Shenzhen Bay business district, which is also the most densely populated area in Nanshan District. The lower revisit rate of shopping centers was close to tourist attractions such as Window of the World and Jinxiu Zhonghua Folk Village. The reason for this phenomenon is due to the large number of shopping centers in the Shenzhen Bay business district, there are more choice for the public, which leads to the lower revisit rate of shopping centers in this area. On the other hand, tourist attractions bring a large number of one-time visitors to shopping centers, which causes the overall revisit rate decreasing.

**Fig 9:**
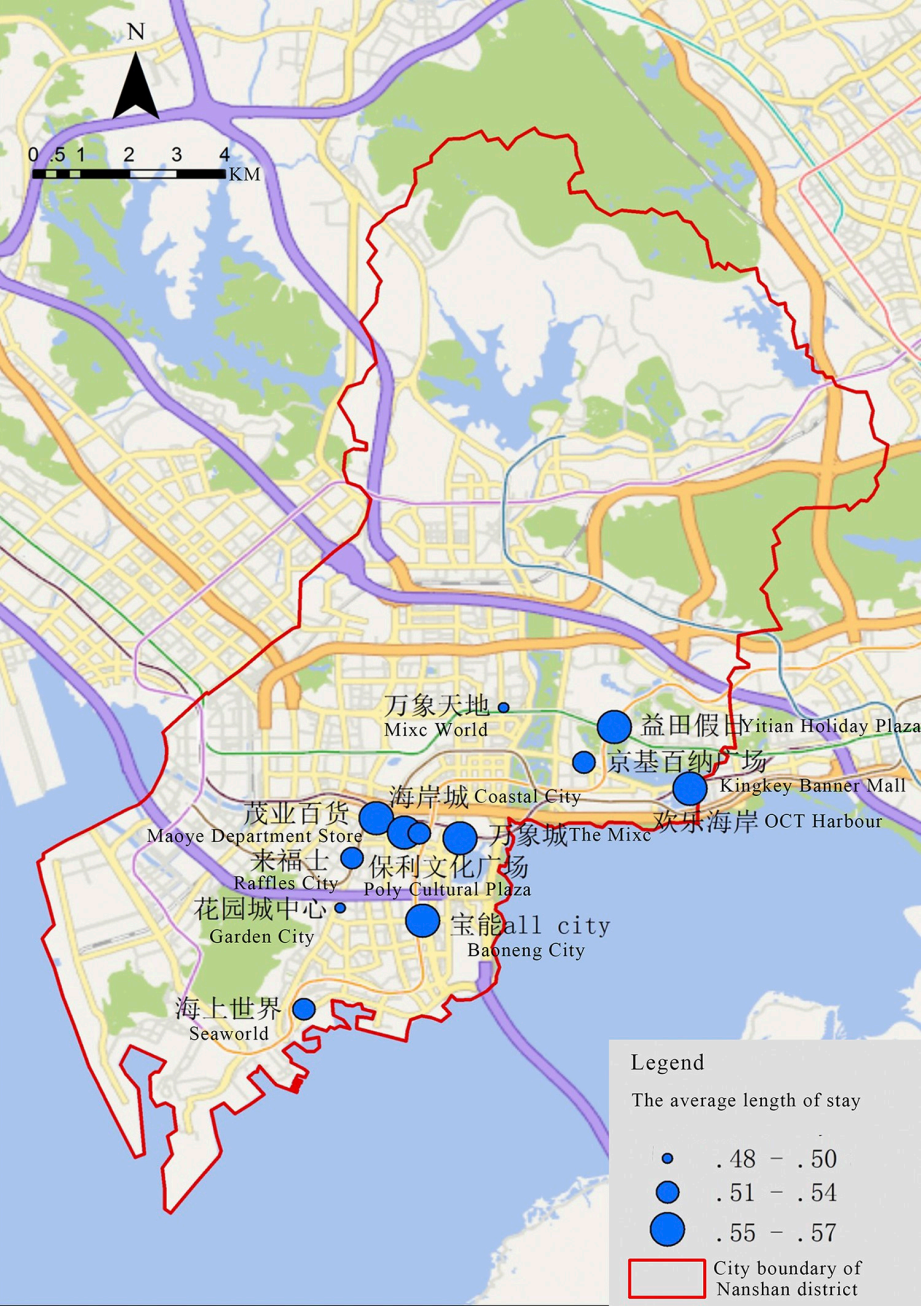
Spatial distribution of the average length of stay of visitors to shopping centers in Nanshan District.

The spatial distribution of the average length of stay of visitors to shopping centers has a clear high-clustering result (Fig 9), forming a high average length of stay circle where Costal City as the core, surrounding with Maoye Department Store and The Mixc. As well as the other business circle that take Window of the World as the core, surrounding with OCT Harbour and Yitian Holiday Plaza. The average length of stay in these two circles is relatively high, while the lower result shopping centers such as Mixc World are more scattered.

**Fig 10:**
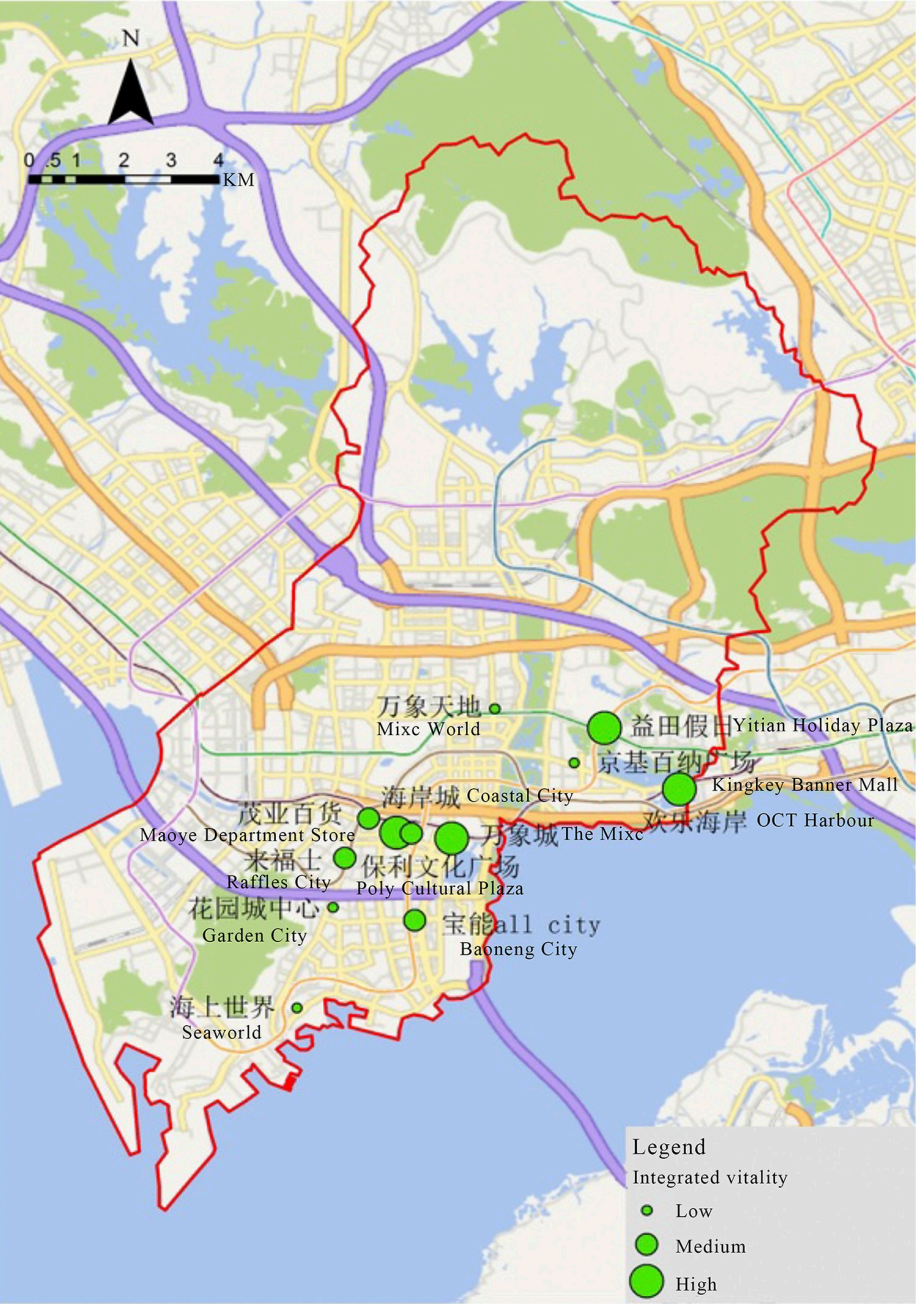
Spatial distribution of integrated vitality of shopping centers in Nanshan District.

In shopping centers, the spatial distribution of integrated vitality is characterized by intermittent high and low results as well as discrete low results. (Fig 10) Maoye Department Store and Raffles City, for example, have average vibrancy in the high vibrancy business circle, namely Coastal City and The Mixc, whereas Seaworld and Mixc World are distinct.

**Fig 11:**
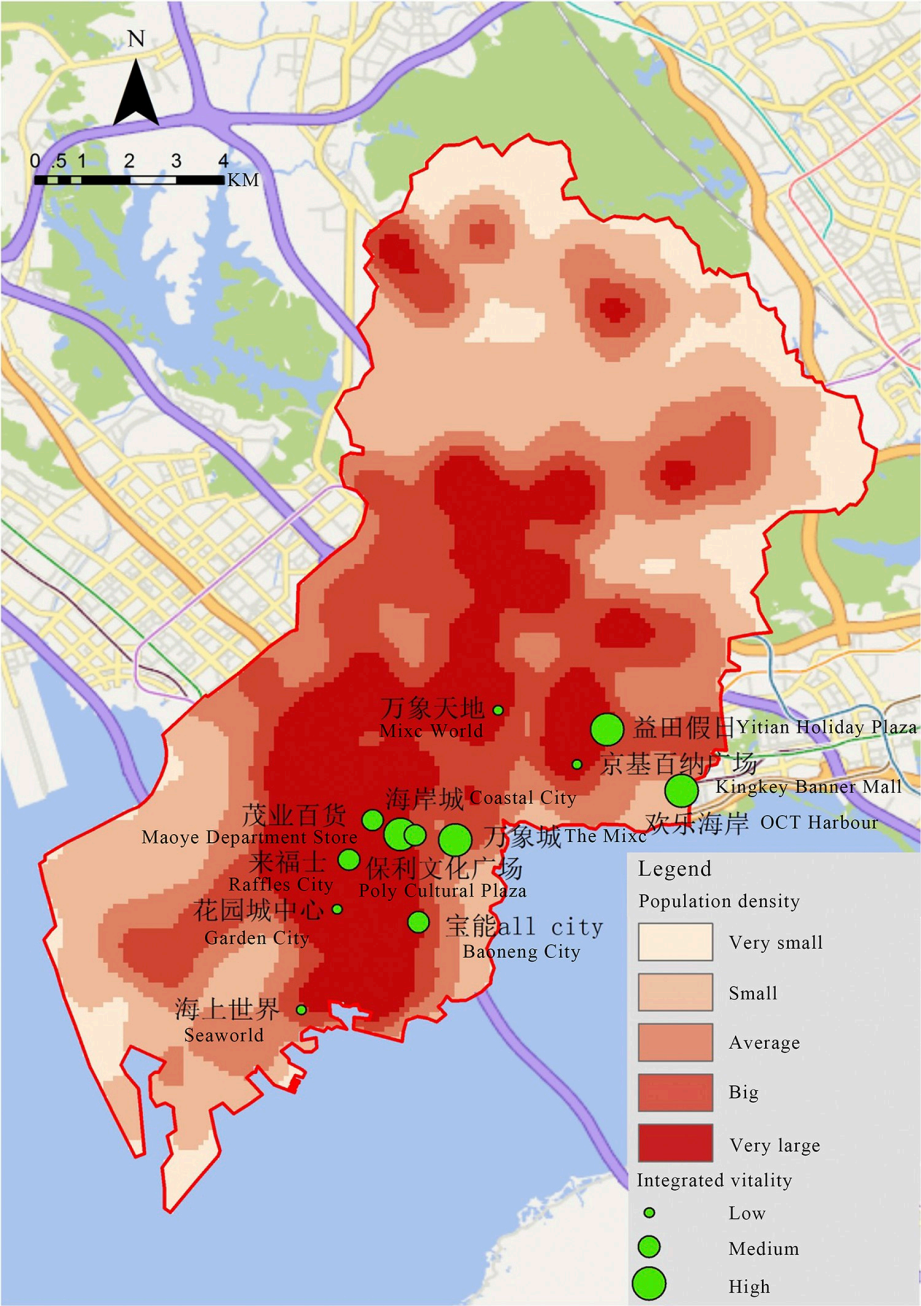
The relationship between the spatial distribution of integrated vitality and population.

We conduct a further analysis that combine the spatial distribution of integrated vitality of shopping centers with the spatial distribution of core population in Nanshan District, the result shows that (Fig 11) the distribution of high vitality shopping centers overlaps with high population density zones, such as Coastal City, Poly Cultural Plaza, and Yitian Holiday Plaza. On the other hand, Mixc World, Garden City, and Kingkey Banner Center are in high density districts but have low vitality. Last but not least, OCT Harbour is unique as it is not located in a high-density area while it has the highest vitality value.

In general, the spatial distribution of shopping centers in Nanshan District has two problems. The first is the unbalanced configuration of shopping centers compared with the population density distribution area and the other is that some shopping centers located in high-population areas are lack of vitality.

### 3.2 Factors influencing the vitality of the built environment of a Shopping Center in Nanshan District

#### 3.2.1 The impact of the built environment on the vitality of shopping centers

Constructing a "15-minute communal living circle" is a requirement for high-quality urban development, in "15-minute communal living circle", all kinds of infrastructure including commercial facilities need to be improved [34]. Moreover, according to “Planning standards for urban planning transport systems”, the maximum transit commute time is 60 minutes for cities with a planned population greater than 5 million. In this article, we study the non-commuting behavior of people visiting shopping centers, and the tolerance for transit travel time is relatively low, so we use a 45-minute transit isochrone circle. Based on the above two points, this article conducts a built environment study based on a 15-minute walking isochrone circle and a 45-minute transit isochrone circle to figure out the factors that could influence the vitality of shopping centers.

As for the selection of factors influencing the built environment, we have reviewed previous research and selected the following factors, including the diversity of sites, the mix of functions, the surrounding facilities, the population and the intensity of construction [26,35–37].

#### 3.2.2 Impact of the built environment on vitality within a 15-minute walking isochrone circle

The 15-minute walking isochrone circle was used to examine the impact of the number of people living within specific walking distance as well as the site characteristics on the vitality of the shopping center. As the comprehensive vitality value of Joy Coast is the highest, the difference with the second ranked shopping center in vitality value is too big, and its low area of 15-minute walking isochronous circle will seriously affect the related indicator values, thus, Joy Coast was recognized as specificity factor, not to be analyzed, observations value is 11. The following table shows the results for other shopping centers (Table 3):

**Table 3:** The relationship between 15-minute walking isochrone circle and shopping center vitality.

Indicators	Comprehensive vitality	Standard errors	Revisit rate	Standard errors	Visitor density	Standard errors	Length of stay	Standard errors
Number of residents	-0.436	0.025	0.733**	0.069	-0.531	0.041	-0.284	0.024
POI mixing degree	-0.040	0.019	0.335	0.051	-0.213	0.031	0.100	0.018
Commercial area	0.630	0.268	-0.254	0.741	0.653*	0.442	0.428	0.262
Green area	-0.173	0.584	0.338	1.612	-0.314	0.963	-0.100	0.571
Land area for public service	0.022	0.208	0.428	0.575	-0.182	0.343	-0.032	0.203
Industrial area	-0.507	0.094	0.417	0.259	-0.535	0.155	-0.834**	0.092
Residential area	0.191	0.306	0.175	0.843	0.024	0.503	0.389	0.298

**. At 0.01 level (double tail), the correlation is significant.

*. At 0.05 level (double tail), the correlation is significant.

In general, the comprehensive vitality is independent of all the factors within the 15-minute walking isochrone circle, however, the revisit rate is significantly and positively correlated with the number of residents, the higher the number of residents within the 15-minute walking isochrone circle, the higher the revisit rate, indicating that the residents around the shopping centers are more likely to form a more regular shopping center visiting pattern, that is the geographic-type vitality. The higher the area of the commercial site, the more concentrated the commercial services are, and the more it will attract people to visit. In contrast, the length of stay is significantly negatively correlated with the area of industrial land, the bigger the area of industrial land, the shorter the length of stay of the visitors.

#### 3.2.3 Impact of the land-use types on vitality within a 45-minute isochrone transit circle

Based on the 45-minute transit isochronous circle, we analyze the influence of the number of residents and land-use types within the 45-minute transit area on the vitality of the shopping center. Although The Mixc shows great comprehensive vitality, it has the smallest 45-minute transit isochronous circle, thus, The Mixc was recognized as specificity factor, not to be analyzed, observations value is 11. The following table shows the results for other shopping centers (Table 4):

**Table 4:** The relationship between 45-minute transit isochronous circle and shopping center vitality.

Indicators	Comprehensive vitality	Standard errors	Revisit rate	Standard errors	Visitor density	Standard errors	Length of stay	Standard errors
Number of residents	-0.065	0.175	0.407	0.176	-0.171	0.302	0.349	0.155
POI mixing degree	0.701*	0.076	0.058	0.076	0.540	0.131	0.578	0.067
Commercial area	0.759*	0.908	-0.580	0.913	0.879**	1.565	0.736*	0.803
Green area	0.888**	0.969	-0.353	0.975	0.885**	1.671	0.776**	0.857
Land area for public service	0.802**	0.873	-0.426	0.878	0.860**	1.505	0.660*	0.772
Industrial area	0.758*	0.782	-0.564	0.786	0.884**	1.347	0.664*	0.691
Residential area	0.772**	1.175	-0.530	1.182	0.880**	2.025	0.667*	1.039

**. At 0.01 level (double tail), the correlation is significant.

*. At 0.05 level (double tail), the correlation is significant.

In general, the factors within the 45-minute transit isochronous circle have a major impact on the shopping center vitality. Integrated vitality is highly significantly and positively correlated with green space, public service and residential land area. The density of pedestrian flow is highly significantly and positively correlated with the area of all five types of land: green space, industrial, residential, commercial, and public service. The length of stay has a highly significant positive correlation with the area of green space. Among them, the correlation between the area of green space and each index is the highest.

**Fig 12:**
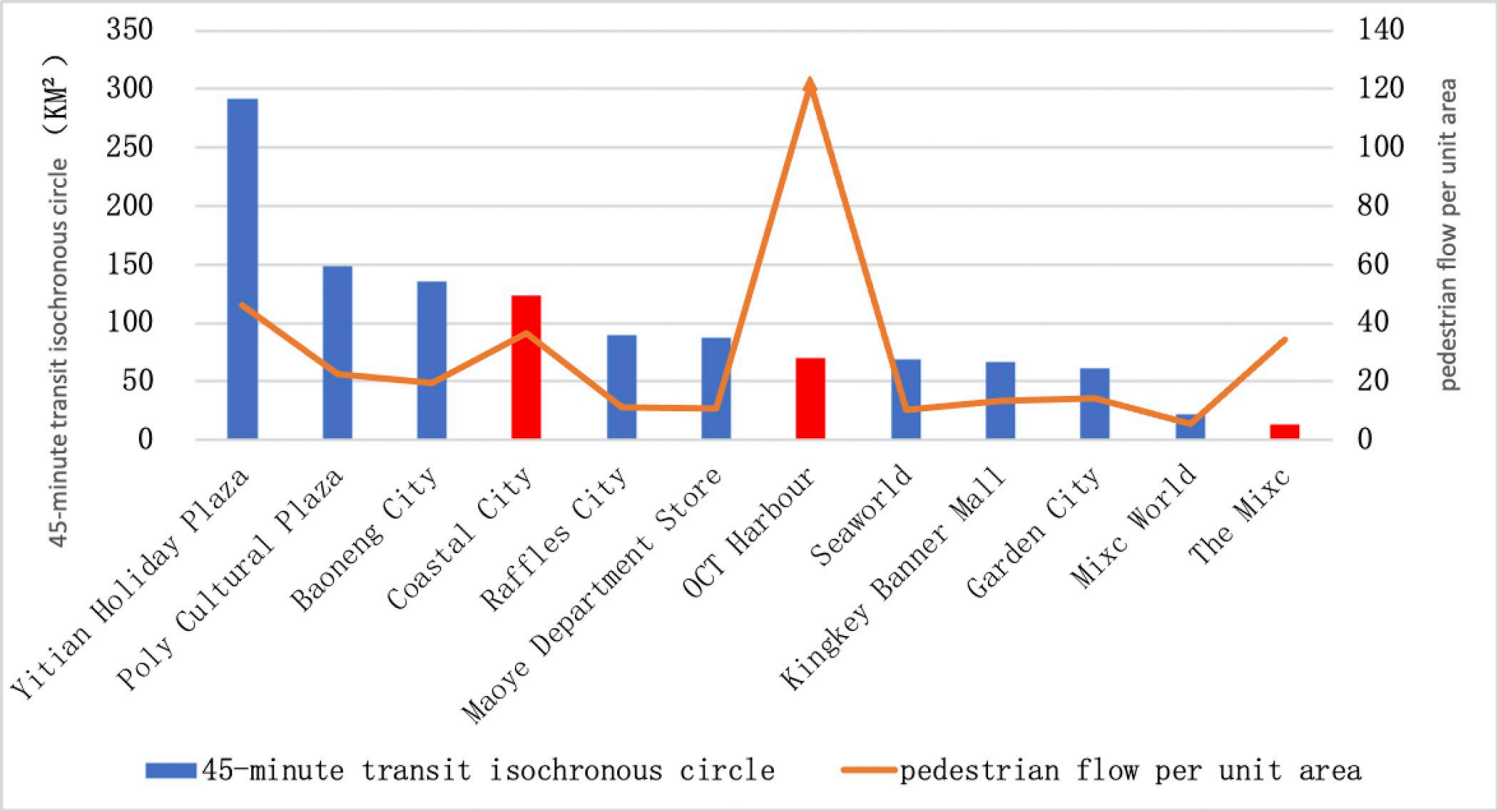
45-minute transit isochronous circle vs. pedestrian flow per unit area for Shopping Centers in Nanshan District.

In addition, since the 45-minute transit isochronous circle is mainly composed of commercial, green space, public service, industrial and residential area, the total area of the above types of land can also represent the area of the 45-minute transit isochronous circle. From Table 4, it is clear that the area of each type of land has a significant impact on the density of pedestrian flow in shopping centers. Therefore, a further analysis of the 45-minute transit isochronous circle of each shopping center compared with the pedestrian flow (Fig 12) shows that except for a few specific factors, the pedestrian flow decreases with the decrease of the 45-minute transit isochronous circle, in other words, the traffic accessibility is also one of the factors affecting the vitality of shopping centers.

**Fig 13:**
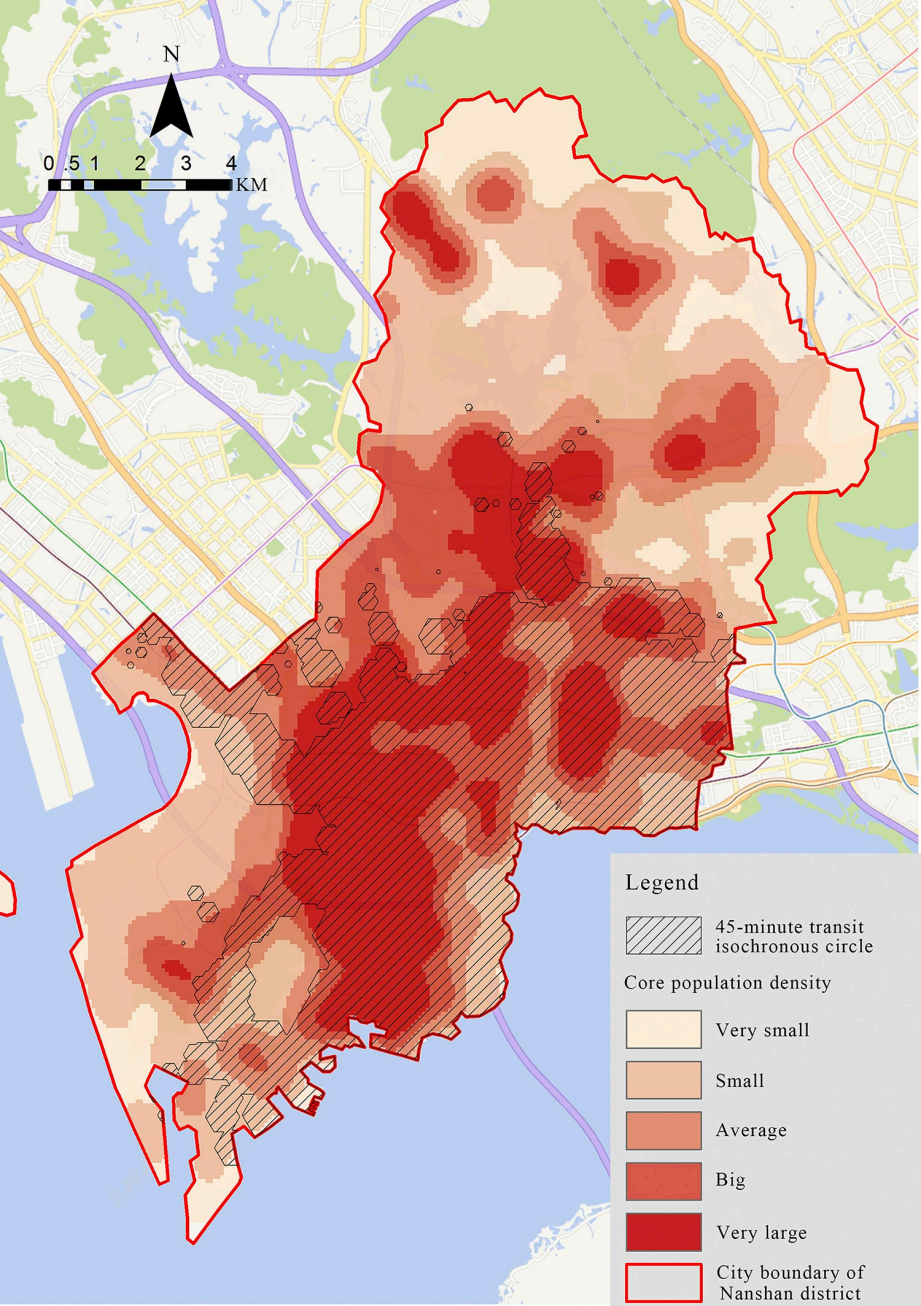
Shopping centers’ 45-minute transit isochronous circle in Nanshan District.

The analysis of the 45-minute transit isochronous circle was continued by overlaying the transit isochronous circle of 12 shopping centers with the density of Nanshan District (Fig 13). It is found that in the high density in the northern of Nanshan District (Xili area), the transit isochronous circle of shopping centers is not fully covered, which indicates that there is a difference in the transportation configuration of shopping centers, the traffic accessibility of Xili area is lower, and there is no large-scale shopping center configuration in this area for the time being.

### 3.3 Field study and validation

A field study was conducted to examine the features of several vitality indicators of different shopping centers under diverse influencing factors. We select the best and worst performing shopping centers in terms of revisit rate, Visitor density, and the length of stay for comparison. (Table 5)

**Table 5:** The shopping centers with the biggest differences in the performance of different vitality indicators.

	Visitor density	Revisit rate	Length of stay
**Highest**	OCT Harbour, Yitian Holiday Plaza,Coastal City,The Mixc	Raffles City,Baoneng City,Maoye Department Store	Yitian Holiday Plaza,Baoneng City,The Mixc,OCT Harbour
**Lowest**	Mixc World	Yitian Holiday Plaza,OCT Harbour	Mixc World

#### 3.3.1 Visitor density

**Fig 14:**
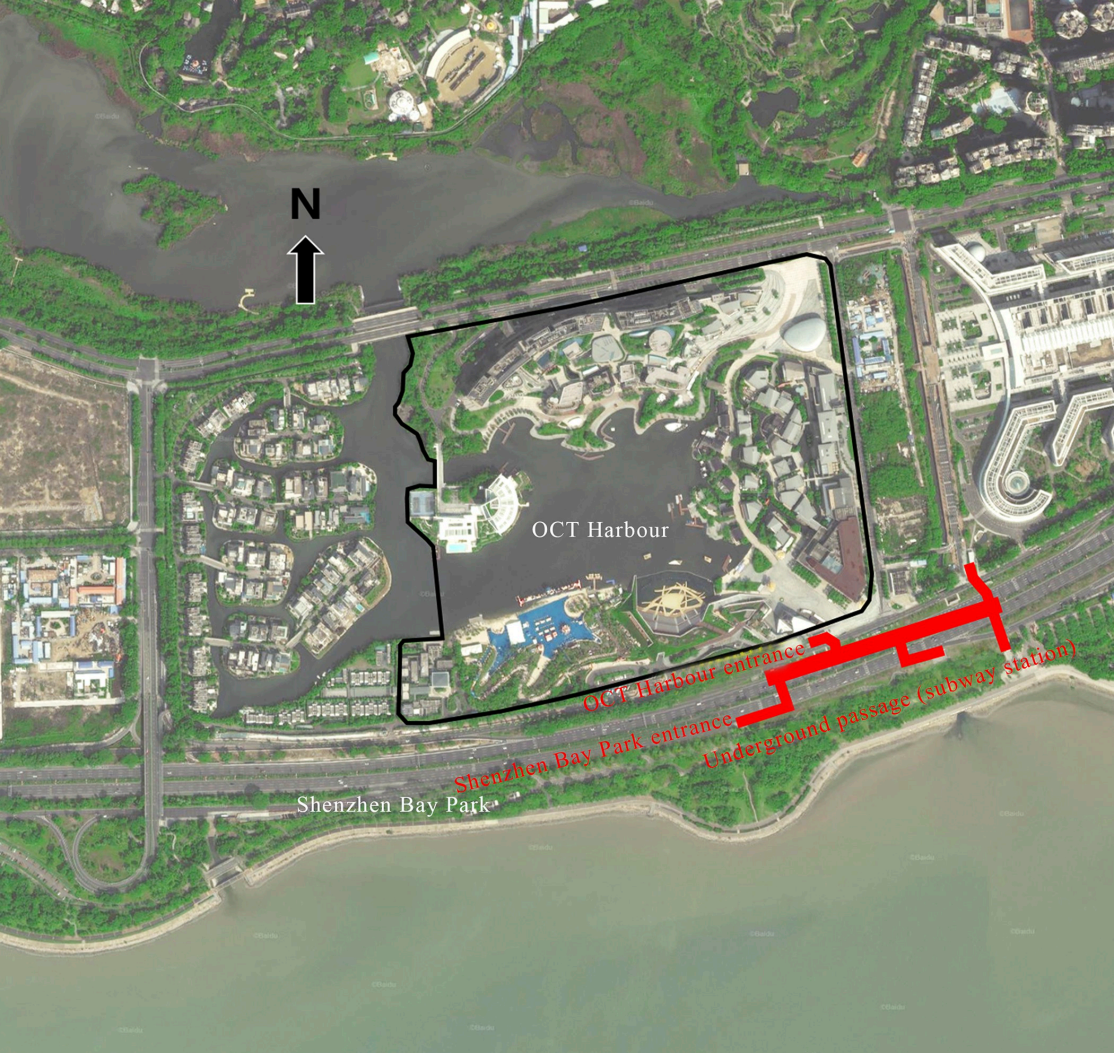
OCT Harbour’s connection to Shenzhen Bay Park.

**Fig 15:**
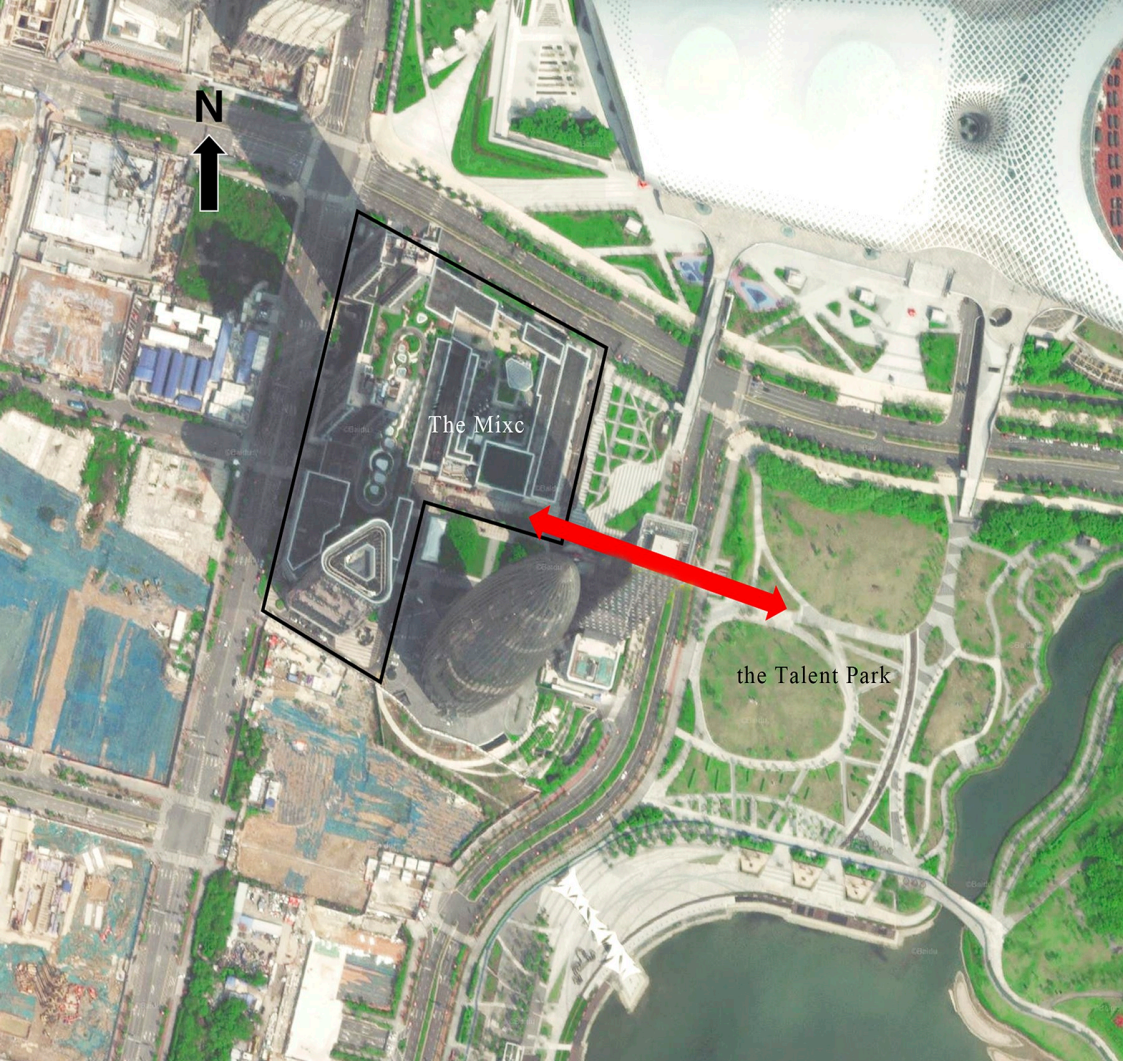
Shenzhen Bay The Mixc’s connection to the Talent Park.

The density of pedestrian flow is highly significantly correlated with the area of green space within the 45-minute transit isochronous circle, and this is also evident from the practical point of view. Happy Coast, the shopping center with the highest pedestrian density, has a large number of green areas and squares and can access to Shenzhen Bay Park within walking distance (Fig 14). From that point, it can increase the attractiveness of the shopping center itself and it is convenient for people from Shenzhen Bay Park to visit OCT Harbour, which ultimately increases the density of pedestrian flow. In addition, the fourth-ranked The Mixc is close to Shenzhen Talent Park, and its junction (Fig 15) is well-connected, so that people from Talent Park can conveniently visit Wanxiang City. The second-ranked Yitian Holiday Plaza has no large green space around it, but it is close to the Window of the World, a large tourist attraction, which also provides Yitian Holiday with a large number of potential visitors.

According to the results of the count of the number of people passing through the junction of The Mixc and the Talent Park from 18:00 to 20:00 on March 21, 2021. It also supports that the Talent Park has a huge attraction effect on The Mixc (Table 6). Talent Park visitors could directly access to the inner streets of The Mixc through Hyde Road 2. There are also a number of experiential and interactive stores and facilities on both sides of the inner streets to attract people to stay, which will increase the vitality of the center.

**Table 6:** Number of people passed at the junction between The Mixc and the Talent Park.

Time slot	Number of Passers
18:00~18:10	95
18:30~18:40	123
19:00~19:10	109
19:30~19:40	87

Mixc World with the lowest result, it was not surrounded by any green areas, parks, or other public space, and there are no other pedestrian flow attractions. The 45-minute transit isochrone circle is the scenterest compared to other shopping centers, which means its traffic accessibility is low. The perimeter of Mixc World is also blocked, which reduces pedestrian flow within walking distance. As a result, Mixc World has the lowest Visitor density, which leads to the lowest comprehensive vitality.

#### 3.3.2 Average length of stay

The average length of stay is also highly correlated with the area of green space within the 45-minute transit isochrone circle. OCT Harbour, which has a length of stay, is adjacent to Shenzhen Bay Park and has a large area of green space in the shopping center itself where Visitor density is also very high. From 18:00 to 21:00 on March 21–22, 2021 (the highest number of visitors to the OCT Harbour), the on-site survey of OCT Harbour showed that there were many leisure spaces and leisure activities in the inner, in that case, the length of stay would be longer.

Mixc World had the lowest average length of stay, for it has the lowest Visitor density compared to other shopping centers. However, an on-site survey of Mixc World on 21–22 February 2021 from 18:00–20:00 (the time period with the highest number of visitors to Mixc World) revealed that it had a number of visitors and leisure activities which is similar to OCT Harbour. The average length of stay can also be referred to as the average active stay. Taking revisit rate into consideration, the revisit rate of Mixc World performs well among all shopping centers. The reason for the opposite results between the revisit rate and the average length of stay in Mixc World, combined with the on-site survey, it can be found that although the shopping center is taking on more and more leisure and entertainment functions, it still has the most essential commercial attributes, and intends to attract more and more visitors, while the shopping center itself provides limited commercial functions. When the number of visitors exceeds its commercial service capacity, it will increase the waiting time of visitors, that will eventually lead to the increase of the overall length of stay. However, the length of stay indicator does not fully represent people’s willingness to stay long enough, so the length of stay indicator commonly used in public service facility vitality studies is not suitable for studying the vitality of shopping centers. The average length of stay of visitors cannot fully reflect the vitality of a shopping center, but only when the shopping center itself can meet the basic needs of all visitors, the length of stay can truly reflect the vitality of a shopping center.

#### 3.3.3 Revisit rate

The results of revisit rate are related to the number of people within walking distance to the shopping center, that is, when the shopping center becomes part of people’s daily life, people are more likely to visit it frequently and eventually form a more regular visiting pattern.

**Fig 16:**
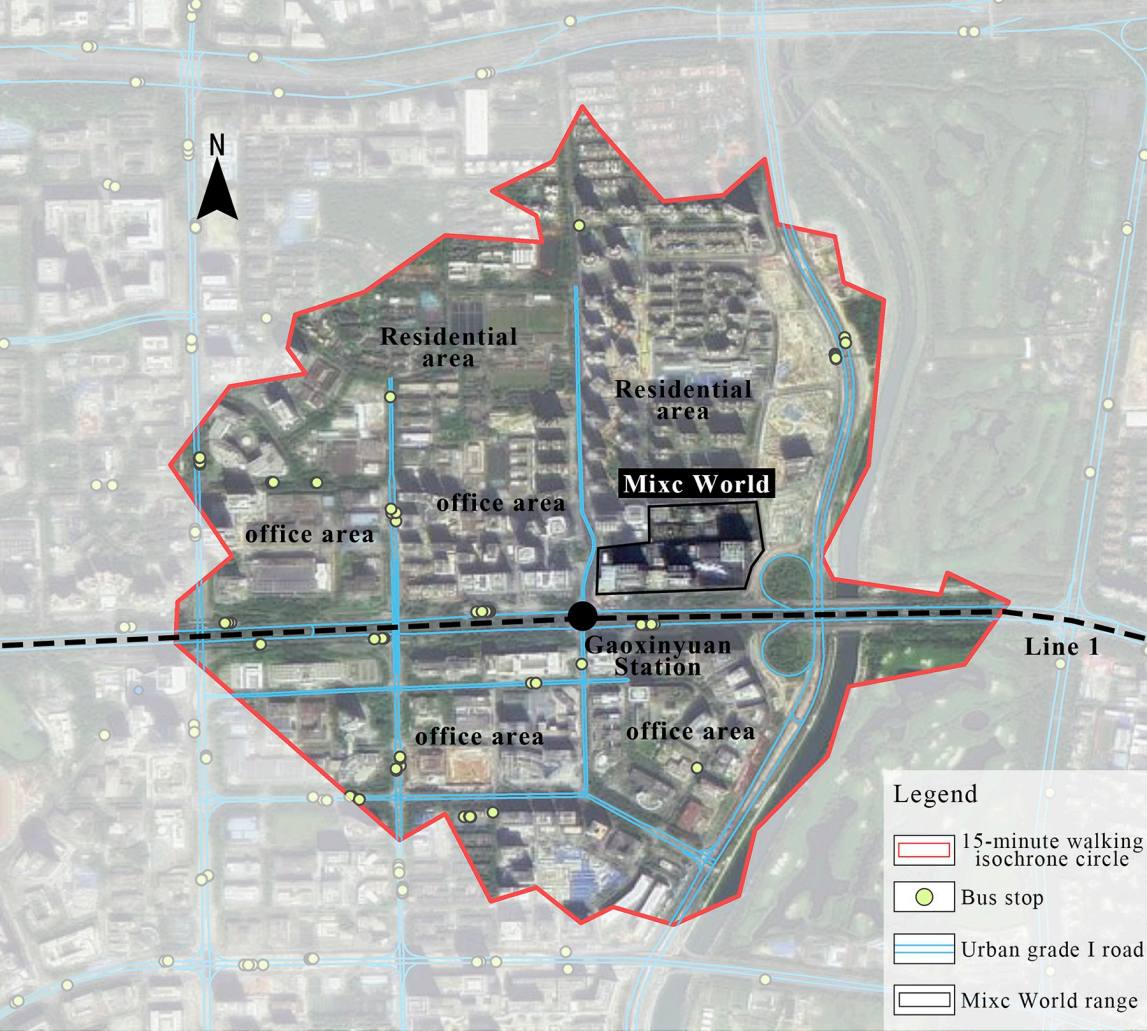
Current situation of building types around Mixc World.

For example, Mixc World, which has the lowest comprehensive vitality, within its 15-minute walking scope are mainly office buildings (Fig 16). The short and regular break time of such users would increase the revisit rate of the shopping center, but the average length of stay is short, which eventually leads to the contrast result between the revisit rate and the average length of stay at Mixc World, and the low revisit rate of OCT Harbour, which has the longest stay, also verifies the above inference.

## 3. Discussion and conclusion

Based on the mobile phone signaling data, the three indicators, namely, “revisit rate”, “visitor density” and “the average length of stay” of twelve large shopping centers in Nanshan District of Shenzhen were used to analyze their vitality characteristics as well as its influencing factors. The results show that: (1) In terms of vitality characteristics, shopping centers in Nanshan District are divided into wide-area type and geo-regional type according to the gathering of people. In terms of the rank of vitality, shopping centers with high vitality are basically wide-area type, with high density of people and long average length of stay, while geo-regional type shopping centers have high revisit rate, that have an obvious hierarchical characteristic of the density of visitors, which has the greatest impact on the vitality of shopping centers. In terms of spatial characteristics, along with the population density distribution, the shopping centers in Nanshan district were constructed unbalanced, some shopping centers located in high population density areas with less vitality. (2) In terms of the influencing factors, traffic accessibility, location adjacent to public spaces and population density within 15-minute walking isochrone circle have significant effects on the vitality of shopping centers in Nanshan District, Shenzhen. Among them, we found that the vitality of wide-area shopping centers is mainly influenced by the land conditions within 45-minute isochrone circle, that is when a shopping center is adjacent to public spaces or tourist attractions, along with its high traffic accessibility, it will increase the number of potential visitors. The vitality of geo-regional type shopping centers is mainly influenced by the number of visitors within 15-minute walking isochrone circle, that is when a shopping center located in a densely populated areas, a regular visiting pattern was formed. In addition, the average length of stay of visitors does not fully reflect the vitality of shopping centers, but only when the shopping centers themselves could meet the basic needs of all visitors.

To conclude, this study takes Nanshan District, Shenzhen as an example, by analyzing refined samples from mobile phone signaling data. It could provide more accurate results compared with traditional survey data and provides more practical arguments for the relevance of influencing factors in combination with field survey results, which has an inspirational effect on the location layout of large shopping centers. The layout of large shopping centers should be analyzed according to the specific needs of the potential population. If the target population is geo-regional type, the site should be in areas with high traffic accessibility and concentrated with large public spaces such as plazas and green areas; if the target population is geo-regional type, the site should be in densely populated areas. In addition, the distribution of large shopping centers should be located as close as possible to the population density, well-overlapped with traffic accessibility, meet the shopping needs of different people, and to achieve a more balanced configuration of large shopping centers.

It is worth noting that the data source for the analysis of the vitality characteristics of the twelve shopping centers is mobile phone signaling data, which could be inaccurate in some ways despite the large amount of data. In the specific comparative analysis of shopping centers, only the shopping centers with the greatest differences in vitality performance were selected for this field research and analysis, making the study conclusions a certain limitation.

## Supporting information

S1 Data (ZIP)
